# Cerebellar Hemangioblastomas in a High-risk Pregnancy: A Case Report and Review of Literature

**DOI:** 10.2174/1573405619666221017114922

**Published:** 2023-04-06

**Authors:** Xue Wang, Yang Liu, Da-Ping Song

**Affiliations:** 1 Department of Neurosurgery, Affiliated Hospital of Southwest Medical University, 25 Taiping Street, Lu Zhou, 646000, China;; 2 Department of Neurosurgery, The Third Hospital of MianYang·Sichuan Mental Health Center, Sichuan, 621000, China;; 3 Department of Pathology, The Third Hospital of MianYang·Sichuan Mental Health Center, Sichuan, 621000, China

**Keywords:** Cerebellar, hemangioblastoma, high-risk pregnancy, case report, GA, CNS

## Abstract

**Introduction:**

Hemangioblastomas are highly vascular benign tumors that may increase in size during pregnancy. The concurrence of cerebellar hemangioblastoma in high-risk pregnancy is extremely rare and the treatment in this situation can be challenged.

**Case:**

Here, we report a case of a 30-year-old woman in the 33rd PW who had experienced a severe headache, dizziness, vomiting, and limb weakness. Cesarean section was performed in the 34th PW, followed by neurosurgery under multidisciplinary discussion.

**Discussion:**

The pathological exam suggested hemangioblastomas. Finally, both the pregnancy and the fetus had a good outcome.

**Conclusion:**

This case emphasizes that the timing of surgery should be determined according to the neurological symptoms of the pregnancy and the gestational age (GA) and condition of the fetus.

## 1 INTRODUCTION

Hemangioblastomas are highly vascular benign tumors that occur either sporadically (66%) or as part of VonHippel-Lindau (VHL) disease (33%) [1, 2]. The incidence of symptomatic cerebellar hemangioblastoma during pregnancy is unknown specifically, with only a few cases reported. There is some pertinent literature reporting the impact of pregnancy on the evolution of hemangioblastoma [3-5]. Besides, the concurrence of this tumor and high-risk pregnancy is extremely rare, and there is no specific recommendation in the literature for the ideal management of this situation.

## 2 PATIENT INFORMATION

**Fig. (1):**
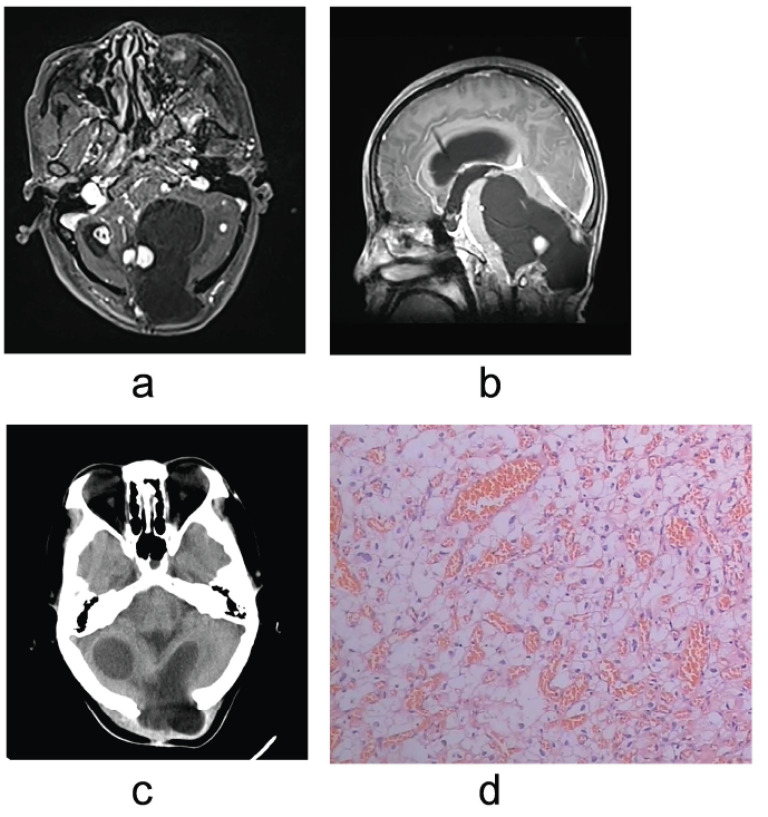
(**a**) and **(b**): A preoperative enhanced MRI showed multiple cystic and solid lesions within both cerebellar hemispheres, resulting in compression of the fourth ventricle. (**c**): A postoperative computed tomography scan performed 5 days after surgery showed a decrease in the size of the cystic lesion, especially in the left cerebellar. (**d**): Hemangioblastoma was verified *via* pathological examination.

A 30-year-old woman was admitted to our hospital in the 33rd pregnancy week (PW). She reported a severe headache accompanied by dizziness, vomiting, and limb weakness in the past two weeks. Besides, she also complained of vision loss which became severe. There was no family history of VHL disease. Besides, her medical history was unremarkable except that she had had three craniotomies for intracranial mass. Ultrasound results were normal and the VHL gene mutation test result was negative. All the results of the histopathologic examination were hemangioblastomas (Fig. **1a**-**d**).

## 3 CLINICAL FINDINGS

On physical examination, alternate motion and finger-to-nose tests were present in the upper limbs, right better than left. Magnetic resonance imaging (MRI) (Fig. **1a**) showed multiple solid nodules on the wall of a large, cystic lesion in the left cerebellar measuring 5.8x5.2cm, compressing the fourth ventricle. At the same time, another cystic lesion was found behind the nodular. The right cystic lesion was measured at 2.6cm in diameter.

## 4 THERAPEUTIC INTERVENTION

Two days later, the obstructive hydrocephalus was aggravated, along with deepening consciousness. To relieve intracranial pressure and allow the pregnancy to progress as closer to the term as possible, a right ventricle puncture and drainage were performed under local anesthesia.Taking the time restriction of the ventricular drainage tube and the safety of the fetus into account, a cesarean section was performed at 34 PW based on multidisciplinary discussions and the consent of her family members. After three days, the cerebellar lesions were partly excised and histopathologic examination (Fig. **1c**) confirmed that they were hemangioblastomas, while the rest of them were recommended to be treated with a gamma knife. A postoperative computed tomography scan Fig. (**1b**) showed the cystic lesions were smaller than before, while the solid nodules were partially removed.

## 5 FOLLOW-UP AND OUTCOMES

After the surgery, her symptoms were relieved obviously and she was discharged symptom-free on the 10th postoperative day. There was no neurological deficit.

## 6 PATIENT PERSPECTIVE

The patient was satisfied with the treatment outcomes and said the fetus was growing well.

## 7 RESULTS AND DISCUSSION

Hemangioblastomas are classified as Grade I meningeal tumors of uncertain origin according to the World Health Organization (WHO) classification [6, 7]. The most commonly affected intracranial location was the cerebellum, which accounts for 93.3% of 30 cases in patients with central nervous system (CNS) hemangioblastomas [8]. In cerebellar hemangioblastomas, ataxia, discoordination, and increased intracranial pressure were often presented [8]. The lesions that occurred in the cerebellum were usually multifocal and cystic, accompanied by some solid nodular that were inserted into the cystic wall [9, 10]. Severe symptoms caused by tumors were considered to be secondary to changes in peripheral tissue pressure or bleeding, mainly including headache, dizziness, vomiting, abnormal behavior, and epilepsy [11, 12]. However, it seems that pregnancy may accelerate the rapid progression of tumors [3]. Although the impact of pregnancy on sporadic hemangioblastomas remains to be poorly understood, several hypotheses have been proposed. For example, vascular engorgement may cause rapid enlargement of tumors due to the increased maternal blood volume [3]. Simultaneously, cerebral edema and amplified neurological symptoms may appear because of the expansion in vascular beds and a decrease in plasmaosmolality and albumin concentration [13]. In addition to this hemodynamic change, hormone effects on tumor growth rates also exist, which are mainly mediated by hormonal receptors [3]. The last hypothesis suggests that both placental growth factor (PlGF) and the vascular endothelial growth factor receptor 1 (VEGFR-1) are expressed at high levels during pregnancy, which have both been reported as part of various pathological processes of angiogenesis and tumorigenesis, causing the formation and growth of peritumoral edema and cysts. In our case, the patient's consciousness worsened suddenly, which was considered to be the result of aggravating obstructive hydrocephalus. Due to the concurrence of high-risk pregnancy and symptomatic cerebellar hemangioblastomas, the treatment could be challenging [13]. The literature indicated that symptomatic treatment was the first choice for pregnant patients with CNS hemangioblastoma. If necessary, urgent tumor resection was possible with careful maternal and fetal monitoring. Besides, it was of great importance to do well in preoperative preparations, intraoperative monitoring, and postoperative management [14]. There are also several case reports sharing some experiences for treatment under similar circumstances. For high-risk pregnancies, Erdogan B *et al*. recommended a conservative temporizing approach that cyst drainage was performed to deliver at full term, including an ommaya reservoir and periodic percutaneous drainage. However, the mass effect of the solid tumor mass seemed to be a contraindiction [13]. In our case, considering that premature delivery may result in fetal dysplasia, we performed a ventricular puncture and drainage to reduce intracranial pressure and extended the gestational period as long as possible. But after a week, neurological symptoms worsened, with a high risk of intrauterine distress and fetal death. A cesarean section was successfully performed immediately, followed by tumor resection. For high-risk pregnancies in the third trimester, some authors recommended urgent tumor resection when neurological deficits still progressed after symptomatic therapies [2, 15, 16]. However, the difference in our case is that cesarean section was performed firstly for the following reasons: 1) general anesthesia, persistent operation time, and blood pressure fluctuation are dangerous to the fetus during the tumor resection surgery; 2) the fetal nutrition condition was poor due to the pregnancy was suffered severe vomiting and unable to eat, so termination of pregnancy was needed. Therefore, between the tumor resection and cesarean section, which one should be carried out in advance? Although symptomatic treatment for pregnancy with CNS hemangioblastoma was generally recommended [2, 14], the timing of surgery should be determined according to the neurological symptoms of the pregnancy and the GA of the fetus. Specific quantitative indicators need to be further studied with more similar cases.

## CONCLUSION

In summary, we report a rare case of a 30-year-old woman with a high-risk pregnancy and cerebellar hemangioblastomas. Besides, the timing of surgery should be determined according to the neurological symptoms of the pregnancy and the GA and condition of the fetus. Finally, multidisciplinary discussions were instructive in reducing risks to some extent. In our case, both the pregnancy and the fetus had a good outcome ultimately, supplying some guidance for similar cases in the future.

## Data Availability

Not applicable.

## LIST OF ABBREVIATIONS

CNS: Central Nervous System
GA: Gestational Age
MRI: Magnetic Resonance Imaging
PIGF: Placental Growth Factor
PW: Pregnancy Week
VEGFR-1: Vascular Endothelial Growth Factor Receptor-1
VHL: von Hippel-Lindau
WHO: World Health Organization
